# Caregiver Acceptability of Mobile Phone Use for Pediatric Cancer Care in Tanzania: Cross-sectional Questionnaire Study

**DOI:** 10.2196/27988

**Published:** 2021-12-08

**Authors:** Kristin Schroeder, James Maiarana, Mwitasrobert Gisiri, Emma Joo, Charles Muiruri, Leah Zullig, Nestory Masalu, Lavanya Vasudevan

**Affiliations:** 1 Department of Pediatric Oncology Duke University Medical Center Durham, NC United States; 2 Department of Oncology Bugando Medical Centre Mwanza United Republic of Tanzania; 3 Duke Global Health Institute Durham, NC United States; 4 Department of Pediatrics Vanderbilt University Medical Center Nashville, TN United States; 5 Department of Population Health Sciences Duke University Durham, NC United States; 6 Durham Veterans Affairs Center of Innovation to Accelerate and Practice Transformation Durham, NC United States; 7 Department of Family Medicine and Community Health Duke University Durham, NC United States

**Keywords:** mHealth, literacy, smartphone use, developing countries, pediatric cancer, cancer, pediatrics, children, parents, caregivers, mobile health, smartphone, SMS, education, knowledge transfer, communication

## Abstract

**Background:**

There is a 60% survival gap between children diagnosed with cancer in low- and middle-income countries (LMICs) and those in high-income countries. Low caregiver knowledge about childhood cancer and its treatment results in presentation delays and subsequent treatment abandonment in LMICs. However, in-person education to improve caregiver knowledge can be challenging due to health worker shortages and inadequate training. Due to the rapid expansion of mobile phone use worldwide, mobile health (mHealth) technologies offer an alternative to delivering in-person education.

**Objective:**

The aim of this study is to assess patterns of mobile phone ownership and use among Tanzanian caregivers of children diagnosed with cancer as well as their acceptability of an mHealth intervention for cancer education, patient communication, and care coordination.

**Methods:**

In July 2017, caregivers of children <18 years diagnosed with cancer and receiving treatment at Bugando Medical Centre (BMC) were surveyed to determine mobile phone ownership, use patterns, technology literacy, and acceptability of mobile phone use for cancer education, patient communication, and care coordination. Descriptive statistics were generated from the survey data by using mean and SD values for continuous variables and percentages for binary or categorical variables.

**Results:**

All eligible caregivers consented to participate and completed the survey. Of the 40 caregivers who enrolled in the study, most used a mobile phone (n=34, 85%) and expressed high acceptability in using these devices to communicate with a health care provider regarding treatment support (n=39, 98%), receiving laboratory results (n=37, 93%), receiving reminders for upcoming appointments (n=38, 95%), and receiving educational information on cancer (n=35, 88%). Although only 9% (3/34) of mobile phone owners owned phones with smartphone capabilities, about 74% (25/34) self-reported they could view and read SMS text messages.

**Conclusions:**

To our knowledge, this is the first study to assess patterns of mobile phone ownership and use among caregivers of children with cancer in Tanzania. The high rate of mobile phone ownership and caregiver acceptability for a mobile phone–based education and communication strategy suggests that a mobile phone–based intervention, particularly one that utilizes SMS technology, could be feasible in this setting.

## Introduction

Each year, low- and middle-income countries (LMICs) account for over 85% of the 400,000 newly diagnosed pediatric cancer cases [1]. Survival rates of these cases range from 5% to 25% in LMICs to over 80% in high-income countries (HICs) [2,3]. Almost one-third of the survival difference can be attributed to treatment abandonment, defined as the failure to initiate or sustain treatment during 4 or more successive weeks [3]. Although health system barriers underlie various causes of treatment abandonment, patient-level barriers also contribute to this phenomenon. For instance, caregiver interviews in LMICs identified limited cancer awareness at the community level and treatment knowledge as critical factors influencing treatment abandonment [4-6]. Hence, in addition to health system strengthening efforts, we need innovative strategies to reduce patient-level barriers and improve survival outcomes for children with cancer in LMICs.

Bugando Medical Centre (BMC) is a tertiary, urban hospital located in Mwanza, Tanzania, and it is one of the three cancer treatment centers in the country. The catchment area comprises 18 million people, and an estimated 1100 new pediatric cases of cancer are diagnosed annually (age <18 years) in this region [7-9]. Of these children, only 20% present for clinical diagnosis and treatment, and over 40% abandon treatment prior to completion. In interviews at BMC, caregivers identified challenges of inadequate care coordination and limited communication between pediatric cancer providers, patients, and themselves as reasons for treatment abandonment [10,11]. Among caregivers of children diagnosed with cancer, fewer than 20% knew their child’s diagnosis or that potentially curative treatment was available for childhood cancer [11]. Owing to limited human resources in many LMIC settings, in-person education and individualized patient navigation and follow-up is often neither feasible nor cost effective [12]. Hence, identification and implementation of alternative modalities of patient education and support in LMIC settings may facilitate caregiver education and support for treatment completion.

With increasing global rates of cellular subscriptions, mobile phones may offer an alternative modality of communication for patient-facing interventions to improve cancer education and treatment support. According to the World Bank, mobile phone subscription rates in Tanzania in 2019 were as high as 82%, reflecting an increase compared to previous years [13]. In recognition of this growing digital technology landscape, the Tanzanian Ministry of Health, Community Development, Gender, Elderly and Children established the National Digital Health Strategy 2019–2024 [14]. This national strategy seeks to establish a strong digital health infrastructure within health systems to promote the quality of health service delivery and support improved health outcomes. Moreover, investments in patient-facing mobile health (mHealth) strategies, in parallel, could help reduce gaps in pediatric oncology care in Tanzania and bolster the evidence base for these technologies in reducing treatment abandonment in LMICs.

The recent World Health Organization digital health guidelines encouraged the use of mobile devices for patient-facing interventions and targeted client communication in particular [15]. Underlying this guideline is a key principle for digital development, which highlights the need to understand the existing ecosystem, including “technology infrastructure and other factors that can affect an individual’s ability to access and use a technology or to participate in an initiative” [16]. However, mobile phone ownership and use patterns among caregivers of pediatric patients with cancer and their acceptability toward using these devices for communication related to health education and care coordination are not well established. To bridge this gap, we conducted a cross-sectional survey assessing caregiver patterns of mobile phone ownership and use, as well as the acceptability of mobile phone use for improving caregiver education, provider-patient communication, and care coordination at BMC in the context of pediatric cancer care.

## Methods

### Study Setting

BMC is a 950-bed consultant hospital located in Mwanza, Tanzania. It is one of the three cancer treatment centers in the country, and the only oncology referral center for the Lake Zone of Tanzania. BMC reports more than 200 newly diagnosed pediatric cancer cases each year [8].

### Study Design and Participants

In July 2017, a cross-sectional survey was conducted among a purposive sample of caregivers of children aged <18 years who were diagnosed with cancer at BMC. All caregivers who were seen in either the inpatient or outpatient setting during the study period were approached for participation in the study. Only one caregiver per patient completed a survey. Informed consent and survey completion was done in either Swahili or English, based on the participant’s language preference. Adult participants provided written informed consent. For participants who self-identified as unable to read, we obtained verbal consent with thumbprint in the presence of a literate witness per institutional standards.

### Survey Questions and Administration

A 26-question survey instrument to elicit descriptive data on patterns of mobile phone ownership and use was previously developed, translated into Swahili, and pilot-tested in the Tanzanian population [17]. Survey domains include mobile phone ownership, technology literacy, and perceived acceptability for digital health interventions. In this study, the section on intervention acceptance was further tailored to include specific pediatric cancer use cases. Participants independently completed the survey. For those who self-identified as unable to read, a patient navigator read the questionnaire aloud and recorded the responses from the caregiver. Surveys were completed in a private room to ensure confidentiality of responses. All surveys were completed on paper, and the responses were stored in a secured office at BMC.

### Statistical Analysis

Statistical analysis was conducted using Excel (version 16; Microsoft Corporation). Descriptive statistics were generated from the survey data using mean and SD values for continuous variables and percentages for binary or categorical variables.

### Ethics Approval

The study was reviewed and approved by the National Institute for Medical Research in Tanzania (NIMR/HQ/R.8a/Vol. IX/3096), the Ethics Committee at BMC (CREC/292/2018), and Duke University Center Institutional Review Board (PRO00094010).

## Results

### Overview

All eligible caregivers who were approached (N=40) agreed to participate in the study. Survey findings related to mobile phone ownership and use are described in Table 1.

**Table 1:** Mobile phone ownership and use among caregivers (N=40) of pediatric patients with cancer at Bugando Medical Centre, Tanzania.

Characteristics		Value, n (%)
**Do you use a mobile phone?**		
	Yes	34 (85)
	No^a^	6 (15)
**What type of mobile phone do you use?**		
	Basic phone (non–touch screen)	31 (91)
	Android Smartphone	3 (9)
**Who owns the mobile phone you use?**		
	Self	33 (97)
	Spouse (husband or wife)	1 (3)
**Do you share your mobile phone with others?**		
	Yes	6 (18)
	No	28 (82)
**With whom do you share your mobile phone?^b^**		
	Spouse (husband or wife)	2 (33)
	Someone in the community	1 (17)
	Other	3 (50)
**Do you use multiple SIM^c^** **cards with your mobile phone?**		
	Yes	21 (62)
	No	13 (38)
**Which of the following mobile networks do you use?^d^**		
	Airtel	22 (65)
	Halotel	6 (18)
	TTCL^e^	2 (6)
	Tigo	5 (15)
	Vodacom	26 (76)
**For what purpose do you use a mobile phone?**		
	Personal use only	13 (38)
	Work and personal use	21 (62)

^a^Additional questions only asked of participants who reported using a mobile phone.

^b^Asked only if participants previously answered “Yes” to sharing their phone.

^c^SIM: subscriber identification module.

^d^Can have multiple networks.

^e^TTCL, Tanzania Telecommunications Company Limited.

### Mobile Phone Ownership

Of the 40 participating caregivers, the majority (n=34, 85%) reported mobile phone use. Of these, 97% (33/34) owned mobile phones, and 3% (1/34) reported their spouse as the primary owner of the mobile phone. Most caregivers (31/34, 91%) owned mobile phones that did not have smartphone capabilities. Vodacom and Airtel were the two most used cellular networks, reported by 76% (26/34) and 65% (22/34) of respondents, respectively.

### Technology Literacy

We assessed survey respondents’ technology literacy pertaining to their mobile devices (Table 2). All caregivers with mobile phones reported being able to receive phone calls. A majority of respondents reported being able to view and read a text message (25/34, 74%), but fewer participants reported being able to compose text messages. About 1 in 2 caregivers (18/34, 53%) knew how to take and send a picture via a cell phone, and 47% (16/34) knew how to watch videos.

**Table 2:** Technology literacy among caregivers of pediatric patients with cancer who own mobile phones (n=34) at Bugando Medical Centre, Tanzania.

Characteristics		Value, n (%)
**Turn phone on** **or** **off**		
	Able	34 (100)
	Not able	0 (0)
**Charge phone**		
	Able	30 (88)
	Not able	4 (12)
**Make phone calls**		
	Able	33 (97)
	Not able	1 (3)
**Receive phone calls**		
	Able	34 (100)
	Not able	0 (0)
**Type using the mobile phone keyboard (** **ie,** **to compose a text message or email)**		
	Able	16 (47)
	Not able	18 (53)
**Send a text message**		
	Able	22 (65)
	Not able	12 (35)
**Open and read a text message**		
	Able	25 (74)
	Not able	9 (26)
**Take pictures**		
	Able	18 (53)
	Not able	16 (47)
**Watch video**		
	Able	16 (47)
	Not able	18 (53)
**Charging Phone**		
	Never	22 (67)
	Sometimes	3 (9)
	Always	8 (24)
	Unclear	1 (3)
**Network Connectivity (ie, no signal, dropped calls, etc)**		
	Never	22 (65)
	Sometimes	3 (9)
	Always	9 (26)
**Browse the internet**		
	Able	4 (12)
	Not able	30 (88)
**Use an installed** **app** **(eg, WhatsApp)**		
	Able	4 (12)
	Not able	30 (88)
**Download and install** **apps**		
	Able	5 (15)
	Not able	29 (85)
**Make monetary transactions**		
	Able	16 (47)
	Not able	18 (53)
**Change phone settings (eg, brightness of screen)**		
	Able	17 (50)
	Not able	17 (50)
**Phone theft or loss**		
	Never	16 (47)
	Sometimes	14 (41)
	Always	4 (12)

### Perceived Needs

**Figure 1:**
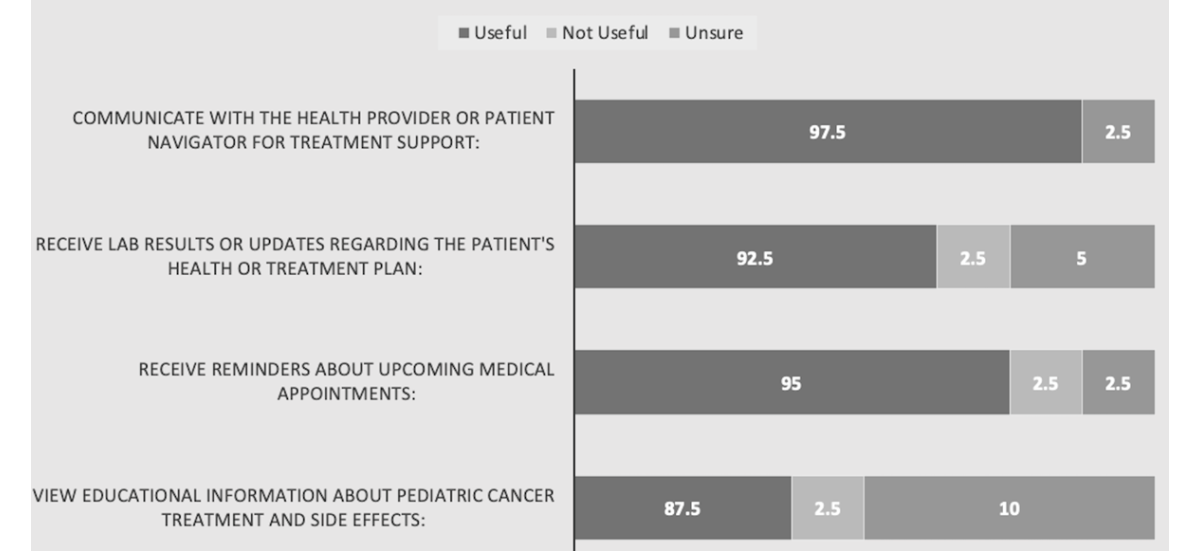
Caregiver acceptability for mobile phone use in pediatric cancer care.

Caregiver responses to the utility of implementing mobile technology in the treatment of pediatric cancer therapy at BMC are illustrated in Figure 1. Of the 40 caregivers, 98% (n=39) thought using mobile technology to communicate with providers would be useful, 95% (n=38) wanted to use mobile technology to receive reminders regarding upcoming appointments, and 88% (n=35) wanted to receive education material and information. Over half (23/40, 58%) of all respondents answered an additional open-ended free-text response question asking what other benefits mobile technology could have in the treatment for their child. Of those, the majority (22/23, 96%) of respondents focused their answer on the potential use of mobile technology to communicate with a medical provider in a time of emergency (ie, febrile illness or severe nausea or vomiting).

## Discussion

### Principal Findings

mHealth interventions have soared in recent history, with over 500 projects implemented in sub-Saharan Africa in the last decade [18-20]. This proliferation of mHealth interventions is due in part to the rapid expansion of mobile phone use and infrastructure worldwide [21]. The majority of caregivers surveyed at BMC owned and used mobile phones and were interested in using these devices to learn and communicate about their child’s cancer treatment. These findings support high feasibility and acceptability for mHealth strategies at BMC to provide targeted information and communication to caregivers of children with cancer, while reducing burden on limited health care resources and personnel. However, additional studies will be needed to confirm the feasibility and acceptability of any future mHealth interventions that are developed for caregivers.

In this study, the proportion of caregivers who reported using a mobile phone (85%) was similar to the national mobile phone subscription rate in Tanzania (82%) [13]. In sub-Saharan Africa, data plans are often inexpensive, and their use is widespread regardless of socioeconomic status [22,23]. Furthermore, investments in mobile phone infrastructure have led to an estimated 93.7% cell tower coverage nationwide, suggesting that an intervention delivered by mobile phone has a high potential reach in Tanzania [24].

Although access to cell coverage is high, many caregivers reported using multiple SIMs with different cellular carriers. Having multiple SIM cards may be a barrier to implementing interventions since other studies have reported challenges with reaching participants when an alternate SIM is in use [25]. However, in Tanzania, mobile phone owners maintain the same telephone number when they switch networks, as part of the Mobile Number Portability Act [26,27]. The high rate of mobile phone ownership and flexibility between networks in Tanzania are important in establishing consistent communication between patients and providers. Our results reveal that the most effective delivery method of content to caregivers in our study setting was via phone calls, as 97% to 100% of respondents that used a mobile phone were capable of making or receiving a phone call.

Although text messaging is a cheaper alternative to voice-based communication in Tanzania, our findings suggest low literacy among caregivers to support a text messaging intervention. We found that text messaging would not be as effective, as only 74% could read a text message and 65% of respondents could send a text message. When faced with the challenges of low literacy rates, *Wazazi Nipendeni*, a text messaging app for pregnant women in Tanzania, added *supporters* and voice-based technology to read the text messages [21,28]. However, in many LMICs, including Tanzania, there is perceived community stigmatization related to pediatric cancer, and having someone other than an immediate family member read or verbalize messages may exacerbate the existing barriers to cancer diagnosis and treatment. Therefore, further research is needed to understand the acceptability of using family or community *supporters* for childhood cancer and whether community-based cancer stigma poses barriers to such a strategy.

In our study, we surveyed caregivers directly to assess their perspectives on the value of a mobile phone–based intervention. Our data suggest high acceptability and desire among caregivers to use mobile phones to communicate with providers, receive lab and appointment reminders, and view educational material related to their child’s cancer diagnosis. An important point to note is that almost all respondents who answered the free-text question regarding other uses of a digital case management system requested a hotline number they could contact in the event of an emergency. Currently, there are no systems in place for a caregiver to contact a trained oncology provider at BMC, and this is likely the situation in other LMIC settings as well. Including end-user participants in the creation and implementation of technologies increases adoption of the intervention, and the idea of using patient-centered feedback in mHealth systems has been a diverging point between successful and unsuccessful implementations [29]. Our results support this claim, as our user-centric approach identified the need of a direct pathway for caregivers to access information from medical providers about their child’s diagnosis and treatment. Including this information in the implementation of future digital platforms will allow us to better care for patients.

Our study sought to evaluate caregiver acceptability of mobile phone use in the global pediatric oncology setting. Of all the initiatives in the 2014 African Strategies for Health mHealth Compendiums, only one focuses on cancer—mEPOC, an app that provides early detection and prevention of oral cancers. There have not been other reports of mHealth in global pediatric oncology [30]. Therefore, this represents an area of need in LMICs, as supporting caregivers of patients with cancer is known to have a positive impact on parent distress and treatment outcomes in HICs [31,32].

### Limitations

This study has several limitations. First, although the survey was previously translated to Swahili and adapted for use in Tanzania, the transcultural adaptation was done for the southern region of Tanzania, whereas our study was completed in the Northeast region of the country, potentially limiting its generalizability [17]. However, the Swahili language used in Tanzania is the same throughout the country, and the domain questions selected used concrete concepts (ie, if the respondent owned a mobile phone), for which regional variations in interpretation would be unlikely. Second, due to the small sample size, we were unable to conduct advanced statistical analyses to assess associations between caregiver characteristics and acceptability. Future planned studies could provide an in-depth assessment of caregiver acceptance by recruiting a larger sample of respondents. Nonetheless, our data suggests that an mHealth intervention at the pediatric cancer department of BMC would be used by caregivers and that it could decrease treatment abandonment via improved communication with providers and patients, clinic reminders, education, and a hotline for emergencies. Given geographical barriers to care in certain parts of Tanzania, especially in rural settings where traveling to health facilities may entail significant time and financial burden, a medical emergency hotline could be of significant benefit for caregivers. Our high rates of population mobile phone use, feasibility, and acceptability of mobile phone intervention delivery are consistent with other chronic disease mHealth research [33]. With cancer being one of the major causes of death from noncommunicable diseases, and with the number of new cases of pediatric cancers rising, it is imperative that we build the evidence base for patient-facing mHealth interventions in this field.

## Abbreviations

BMC: Bugando Medical Centre
HIC: high-income country
LMIC: low- and middle-income country
mHealth: mobile health
